# RNA demethylase ALKBH5 in cancer: from mechanisms to therapeutic potential

**DOI:** 10.1186/s13045-022-01224-4

**Published:** 2022-01-21

**Authors:** Jianwei Qu, Haimeng Yan, Yifan Hou, Wen Cao, Yang Liu, Enfan Zhang, Jingsong He, Zhen Cai

**Affiliations:** 1 Bone Marrow Transplantation Center, The First Affiliated Hospital, School of Medicine, Zhejiang University, Hangzhou, Zhejiang China; 2 Institute of Hematology, Zhejiang University, Hangzhou, Zhejiang China

**Keywords:** m^6^A modification, ALKBH5, RNA demethylation, Cancer, Gene regulation, Therapeutic target

## Abstract

RNA demethylase ALKBH5 takes part in the modulation of N^6^-methyladenosine (m^6^A) modification and controls various cell processes. ALKBH5-mediated m^6^A demethylation regulates gene expression by affecting multiple events in RNA metabolism, e.g., pre-mRNA processing, mRNA decay and translation. Mounting evidence shows that ALKBH5 plays critical roles in a variety of human malignancies, mostly via post-transcriptional regulation of oncogenes or tumor suppressors in an m^6^A-dependent manner. Meanwhile, increasing non-coding RNAs are recognized as functional targets of ALKBH5 in cancers. Here we reviewed up-to-date findings about the pathological roles of ALKBH5 in cancer, the molecular mechanisms by which it exerts its functions, as well as the underlying mechanism of its dysregulation. We also discussed the therapeutic implications of targeting ALKBH5 in cancer and potential ALKBH5-targeting strategies.

## Introduction

**Fig. 1:**
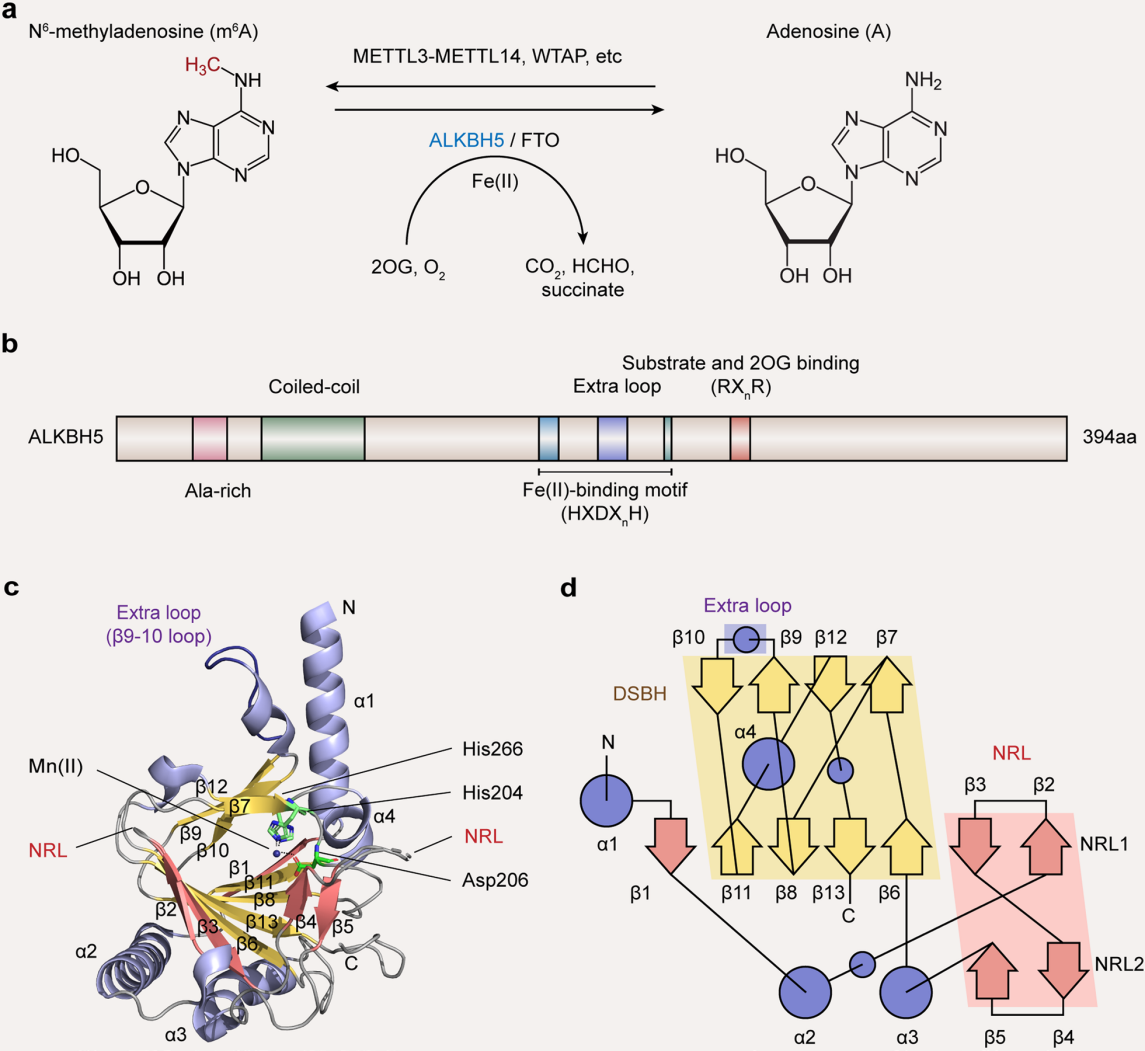
Dynamic regulation of N^6^-methyladenosine (m^6^A) and structure of RNA demethylase ALKBH5. **a** The m^6^A modification is added to the RNA by the methyltransferase complex (writer) composed of the METTL3-METTL14 heterodimer core subunit and additional cofactors such as WTAP. The RNA m^6^A modification could be removed by RNA demethylases (erasers), FTO and ALKBH5 in a reversible manner. **b** Domain structures of ALKBH5. **c** Cartoon representation of the ALKBH5_66–292_ structure (PDB ID: 4NJ4) in complex with Mn(II) [67]. Mn(II) is a substitute for Fe(II). **d** Schematic form of the ALKBH5_66–292_ structure [67]. Arrows represent β-strands and circles represent α-helices. The DSBH is highlighted in yellow, the extra loop (β9-10 loop) in purple and the NRLs in red

More than 170 post-transcriptional modifications in different types of RNA have been reported [1, 2], with N^6^-methyladenosine (m^6^A) being a well-studied RNA modification thus far. m^6^A was firstly described about five decades ago (1970s) and was identified as the commonest internal modification of polyadenylated mRNAs in most eukaryotic species, including mammals [3, 4]. In 2010s, the development of various next-generation sequencing (NGS)-based m^6^A sequencing techniques provided further insights into such epigenetic mark, revealing m^6^A presence in virtually all RNA types, such as mRNAs, ribosomal RNAs (rRNAs), microRNAs (miRNAs), long non-coding RNAs (lncRNAs), circular RNAs (circRNAs) and small nuclear RNAs (snRNAs) [5–12]. m^6^A undergoes a dynamic regulation by RNA methyltransferases (“writers”) and demethylases (“erasers”). The deposition of m^6^A modification into RNAs is catalyzed by the m^6^A methyltransferase complex (MTC) comprising a METTL3-METTL14 heterodimer core alongside other ligands, including WTAP, VIRMA, RBM15A/B, ZC3H13 and HAKAI [13–19]. m^6^A removal from an RNA could be performed by m^6^A demethylases, including fat mass and obesity-associated protein (FTO) and alpha-ketoglutarate-dependent dioxygenase alkB homolog 5 (ALKBH5) (Fig. 1a) [20, 21]. The reversible m^6^A modification contributes to multiple pathways controlling mRNA metabolism and have critical functions in gene regulation. Indeed, m^6^A regulates mRNA splicing [12, 22–25], promotes mRNA nuclear export [26], alters mRNA stability [27–32], increases translation efficiency [30–36] and facilitates non-canonical translation initiation [37]. Most of these effects are controlled by m^6^A “readers”, which selectively interact with m^6^A and exert a regulatory function on the m^6^A-marked mRNA (Table 1). Additionally, m^6^A modifications of non-coding RNAs (ncRNAs) are critical for their expression and functions, with some of them using the same machinery employed by mRNAs for writing, erasing and reading [7, 11, 12, 22, 38–41]. Given its wide-ranging functions in RNA metabolism and gene regulation, m^6^A is unsurprisingly required for numerous biological processes in normal physiology and disease conditions.

**Table 1:** Effects of m^6^A on RNA metabolism

Reader or executor	Cellular localization	Effects on m^6^A RNA	References
YTHDC1	Nucleus	Mediates the splicing and export of methylated mRNAs	[25, 26]
YTHDC2	Cytosol	Regulates mRNA degradation and translation initiation	[32]
YTHDF1	Cytosol	Interacts with initiation factors for facilitating translation initiation of m^6^A-modified mRNAs	[33]
YTHDF2	Cytosol	Promotes the degradation of methylated mRNAs by targeting to P-bodies and recruiting the CCR4–NOT complex	[27, 28]
YTHDF3	Cytosol	Promotes translation in synergy with YTHDF1, and mRNA decay in synergy with YTHDF2	[30, 34]
METTL3	Cytosol	Binds to a few m^6^A mRNAs and promotes translation initiation by mRNA looping	[35, 36]
HNRNPC, HNRNPG	Nucleus	Mediate mRNA splicing	[22–24]
HNRNPA2B1	Nucleus	Regulates m^6^A-dependent microRNA processes and alternative splicing	[12]
IGF2BP1/2/3	Nucleus and cytosol	Protect target mRNAs from degradation in the P-body	[31]
eIF3	Cytosol	Binds to m^6^A sites in the 5′UTRs of mRNAs and promotes cap-independent translation	[37]
HuR	Nucleus and cytosol	Binds to demethylated RNAs and increases RNA stability	[29]

*YTHDC1/2* YTH domain-containing protein ½, *YTHDF1/2/3* YTH domain-containing family protein 1/2/3, *eIF3* Eukaryotic translation initiation factor 3, *METTL3* Methyltransferase-like protein 3, *HNRNPC* Heterogeneous nuclear ribonucleoproteins C1/C2, *HNRNPG* Heterogeneous nuclear ribonucleoprotein G, *HNRNPA2B1* Heterogeneous nuclear ribonucleoprotein A2B1, *IGF2BP1/2/3* Insulin-like growth factor 2 mRNA binding protein 1/2/3, *HuR* Human antigen R

ALKBH5 is a member of the well-conserved AlkB family of non-heme Fe(II)/α-KG-dependent dioxygenases, which mediate the repair of N-alkylated nucleobases by oxidative demethylation [42, 43]. There are nine human AlkB members, including ALKBH1–8 and FTO [44], which have the same 2-oxoglutarate (2OG, also known as α-KG) oxygenase double-stranded β-helix (DSBH) and associated 2OG and iron binding sites, but differ in substrates and functions [42, 45]. This difference exists even between two m^6^A demethylases: while FTO has been demonstrated to demethylate internal m^6^A on mRNAs and U6 RNAs, N^6^, 2-O-dimethyladenosine (m^6^Am) on mRNAs and snRNAs, N^1^-methyladenosine (m^1^A) on tRNAs, 3-methylthymine (m^3^T) on single-stranded DNAs (ssDNAs) and 3-methyluracil (m^3^U) on single-stranded RNAs (ssRNAs) [20, 46–48], ALKBH5 was found to be only responsible for catalyzing the removal of m^6^A on ssRNAs [21]. ALKBH5 appears to be localized to nuclear speckles that contribute to the assembly of mRNA-processing factors, which supports the notion that nuclear nascent RNAs are major substrates of ALKBH5 [21]. ALKBH5 preferentially interacts with the distal 5′ region of coding sequences [49], with specific preference for the consensus sequence Pu[G>A]m^6^AC[A/C/U] (Pu is any purine base) [21]. In normal condition, ALKBH5 shows high expression in the testis and lung, followed by spleen, kidney and liver, with low cardiac and cerebral amounts [21]. Consistently, ALKBH5-deficient mice show impaired spermatogenesis because of abnormal levels of major genes controlling spermatogenic maturation [21].

In addition to spermatogenesis [21, 50], significant biomolecular involvement of ALKBH5 has been demonstrated in osteogenic differentiation [51, 52], heart regeneration [53], embryonic stem cell cardiac commitment [54], brain development [55], post-ischemic angiogenesis [56], ROS-induced DNA damage response [57] and immune response [58–62]. Abnormal expression of ALKBH5 is tightly associated with human pathologies. ALKBH5 was found to be involved in the ossification of the ligamentum flavum [63], Hirschsprung's disease [64] and cerebral ischemia–reperfusion injury [65]. In addition, the role of ALKBH5 in disease has been most extensively studied in the context of cancer. This review summarized recently reported progress in understanding ALKBH5’s structure and up-to-date findings concerning ALKBH5’s roles in cancer, with a main focus on the regulatory effects of ALKBH5 on diverse targets in cancer and how ALKBH5 is dysregulated in cancer. We also highlighted advances in ALKBH5-targeting approaches and the related clinical potential in tumor therapy.

## ALKBH5 structure

The full-length human ALKBH5 has 394 amino acids and contains an active site motif HXDX_*n*_H (X = any amino acid) for Fe(II) binding, RX_*n*_R for 2OG binding and substrate recognition, and an extra loop resulting in preferential interaction with single-stranded over double-stranded nucleic acids (Fig. 1b) [66–69]. In detail, the catalytic core of ALKBH5 exhibits a well-conserved DSBH, in which β6, β8, β11 and β13 form the major β-sheet, whereas β7, β9, β10 and β12 form the minor one (Fig. 1c, d) [67]. The DSBH functions as a scaffold for three Fe(II)-ligating amino acids, including His204, Asp206 and His266 (Fig. 1c), which constitute the well-conserved HXDX_*n*_H motif coordinating metal ions [67]. The 2OG binding site is found in a cavity surrounded by the two DSBH’s β-sheets, with the more open end of this cavity allowing the interaction between substrate and active site [67]. The m^6^A base likely packs against His204 in a pocket comprising Arg130 and Tyr139 [68]. An unanticipated disulfide bond linking Cys230 and Cys267 is found in ALKBH5, which impedes the access of dsDNAs and dsRNAs to its active site [69]. Meanwhile, an extra loop (β9-10 loop) in ALKBH5’s steric clash (amino acids 229–243), causes steric hindrance of dsDNAs (Fig. 1c, d) [67, 68]. Moreover, the nucleotide recognition lid (NRL) outside the DSBH fold, which is further divided into two β hairpin-like loops named NRL1 (β2–3) and NRL2 (β4–5), plays an important role in substrate recognition and catalysis (Fig. 1c, d) [67, 69]. Furthermore, the N-terminus of ALKBH5 has multiple alanine residues and a coiled-coil structure, which may determine its localization [66]. Its C-terminus is likely disordered and comprises Arg–Ser-rich regions, which might control RNA interactions [67]. The knowledge of ALKBH5 structure provides clues for rationally designing specific AKBH5 inhibitors and activators.

## Roles of ALKBH5 in cancer

**Fig. 2:**
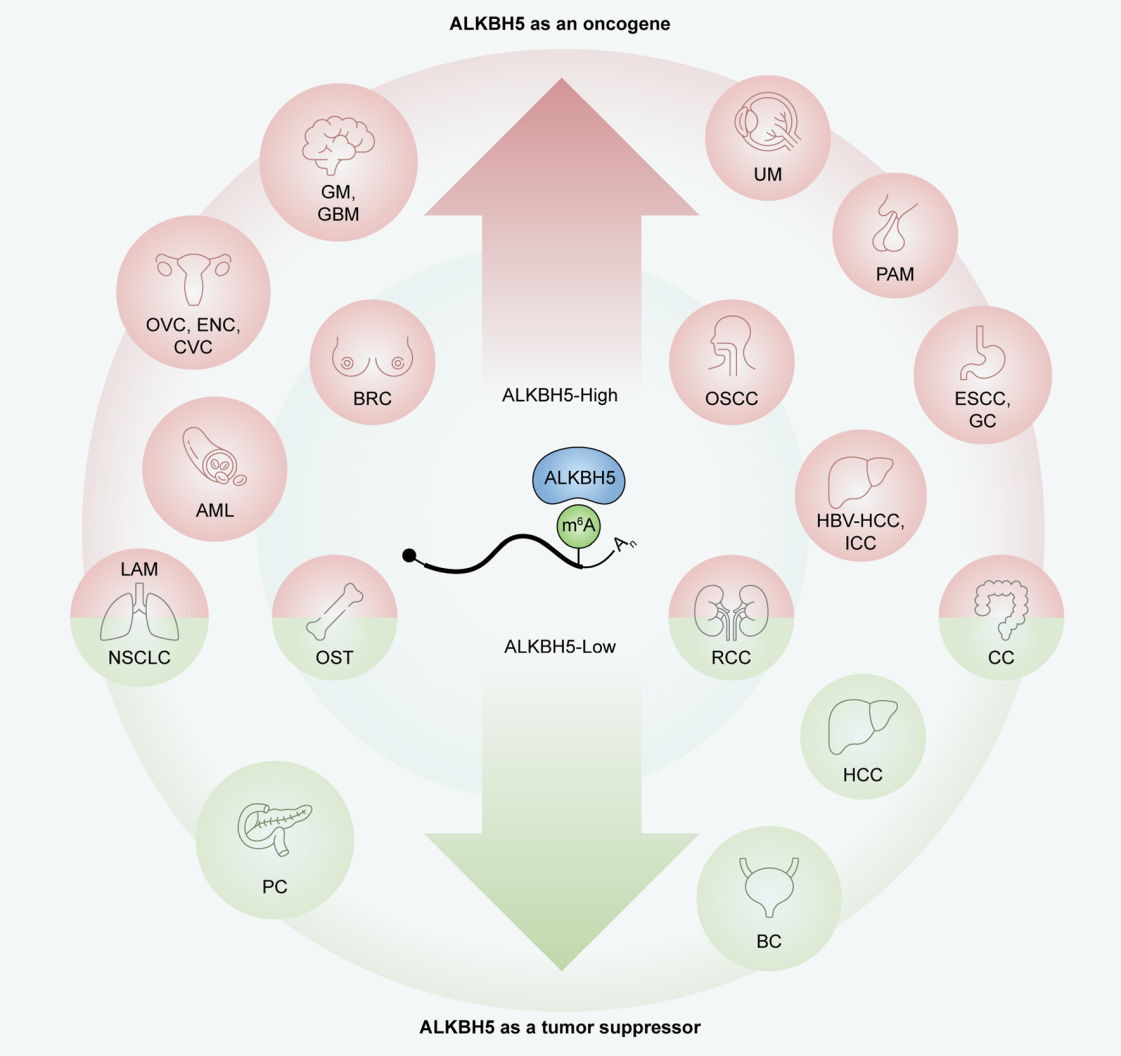
Roles of ALKBH5 in cancers. *GM* Glioma, *GBM* Glioblastoma, *OVC* Ovarian cancer, *ENC* Endometrial cancer, *CVC* Cervical cancer, *BRC* Breast cancer, *AML* Acute myeloid leukemia, *UM* Uveal melanoma, *PAM* Pituitary adenomas, *OSCC* Oral squamous cell carcinoma, *ESCC* Esophageal squamous cell carcinoma, *GC* Gastric cancer, *HBV-HCC* Hepatitis B virus (HBV) related hepatocellular carcinoma, *ICC* Intrahepatic cholangiocarcinoma, *LAM* Lung adenocarcinoma, *NSCLC* Non-small cell lung cancer, *OST* Osteosarcoma, *RCC* Renal cell carcinoma, *CC* Colon cancer, *PC* Pancreatic cancer, *HCC* Hepatocellular carcinoma, *BC* Bladder cancer. ALKBH5 plays an oncogenic role in cancers in red, and plays a tumor-suppressive role in cancers in green; ALKBH5 has controversial roles reported in cancers with both colors

Recent findings suggested ALKBH5 is commonly dysregulated in multiple malignancies, and plays important roles as an m^6^A demethylase. Altered ALKBH5 expression could both promote and suppress carcinogenesis, based on cancer type (Fig. 2; Table 2).

**Table 2:** Roles of ALKBH5 in human malignancies

Role	Cancer type	Upstream regulators	Downstream targets	Molecular mechanisms	Target pathways	Cellular phenotypes	Ref
Oncogene	Breast cancer	HIF-1α/2α	*NANOG*, *KLF4*	Increased mRNA stability	–	Cancer stem cell enrichment	[74, 75]
	–	–	–	–	–	Survival, proliferation, migration	[72]
	Glioma	–	*G6PD*	Increased mRNA stability	Pentose phosphate pathway	Proliferation, energy metabolism	[77]
		*miR-193a-3p*	*AKT2*	Increased mRNA stability and decreased protein degradation	AKT2 pathway	Proliferation, survival	[78]
	Glioblastoma	*FOXM1-As* (lncRNA)	*FOXM1*	Up-regulation of nascent transcripts by HuR	–	Stem cell self-renewal, proliferation	[82]
		–	*CHK1*, *RAD51*, *FOXM1*, *YAP1*	–	–	Radioresistance, invasion	[83]
		*SOX2OT* (lncRNA)	*SOX2*	–	Wnt5a/β-catenin signaling	Temozolomide resistance	[85]
		–	*NEAT1* (lncRNA)	Increased RNA stability	–	Immune evasion	[134]
	Non-small cell lung cancer^a^	–	*TIMP3*	Decreased mRNA stability	–	Proliferation, survival	[87]
		–	*UBE2C*	Increased mRNA stability	–	Proliferation	[88]
		LKB1	*SOX2*, *SMAD7*, *MYC*	Decreased YTHDF2-dependent mRNA decay	–	Proliferation, migration	[89]
	Lung adenocarcinoma	–	*FOXM1*	Increased translation	–	Proliferation, invasion	[90]
		–	*RMRP* (lncRNA)	–	–	Proliferation, migration, invasion, survival	[91]
	Ovarian carcinoma	–	*BCL2*	Increased mRNA stability	Autophagy	Proliferation	[94]
		TLR4	*NANOG*	–	–	Proliferation, survival	[95]
	Acute myeloid leukemia	–	*TACC3*	Increased mRNA stability	P21 and MYC pathways	Proliferation, survival, stem cell self-renewal	[98]
		KDM4C, MLL1/3, MYB	*AXL*	Decreased YTHDF2-dependent mRNA decay	PI3K/AKT/mTOR pathway	Proliferation, survival, stem cell self-renewal	[99]
	Gastric cancer	–	*NEAT1* (lncRNA)	–	–	Invasion, metastasis	[101]
	Colon cancer^a^	–	*NEAT1* (lncRNA)	–	–	Proliferation, migration, survival	[102]
	Intrahepatic cholangiocarcinoma	–	*PDL1*	–	–	Immune evasion, immunotherapy resistance	[132]
	Endometrial Cancer	–	*IGF1R*	–	IGF1R signaling pathway	Proliferation, invasion	[103]
		HIF-1α/2α	*SOX2*	–	–	Stem-like state	[104]
	Renal cell carcinoma^a^	HIF-1α	*AURKB*	Increased mRNA stability	–	Proliferation	[105]
	HBV related hepatocellular carcinoma	HBx	HBx mRNA	Increased mRNA stability	–	Proliferation, migration	[106]
	Osteosarcoma^a^	–	*PVT1* (lncRNA)	Decreased YTHDF2-dependent mRNA decay	–	Proliferation	[107]
	Uveal melanoma	EP300	*FOXM1*	Increased mRNA stability	–	Proliferation, migration, invasion, survival, epithelial-to-mesenchymal transition	[108]
	Pituitary adenomas	HIF-1α	*NANOG*	–	–	Proliferation	[109]
	Esophageal squamous cell carcinoma	–	*CDKN1A*	Decreased mRNA stability	–	Proliferation, migration	[110]
	Oral squamous cell carcinoma	DDX3	*FOXM1*, *NANOG*	–	–	Chemoresistance to cisplatin	[112]
	Intrahepatic cholangiocarcinoma	–	*PDL1*	Decreased YTHDF2-dependent mRNA decay	–	Immune evasion, immunotherapy resistance	[132]
Tumor suppressor	Bladder cancer	–	*CSNK2A1*	Increased mRNA stability	Glycolysis pathway	Proliferation, migration, invasion, chemoresistance to cisplatin	[114]
		–	*ITGA6*	Promotion in translation by YTHDF1/3	–	Cell adhesion, proliferation	[115]
	Pancreatic cancer	–	*KCNK15-AS1* (lncRNA)	–	–	Migration, invasion	[119]
		P53	*PER1*	Increased YTHDF2-dependent mRNA decay	ATM-CHK2-P53/CDC25C signaling pathway	Proliferation, migration, invasion	[120]
		–	*WIF1*	Decreased transcription	Wnt signaling pathway	Proliferation, migration, invasion, chemoresistance to gemcitabine	[121]
	Non-small cell lung cancer^a^	–	*YAP1*, *miR-107/LATS2*	Competitive binding by YTHDF1 and YTHDF2; inhibition of miR-107/LATS2–mediated YAP1 phosphorylation by HuR	–	Proliferation, migration, epithelial-to-mesenchymal transition	[122]
	Osteosarcoma^a^	–	*Pre-miR-181b-1*, *YAP1*	Increased YTHDF2-dependent miRNA decay; Increased YTHDF1-dependent translation	–	Prolifertion, migration, invasion, survival	[123]
	Colon cancer^a^	–	–	–	–	Invasion, metastasis	[124]
	Hepatocellular carcinoma	–	*LYPD1*	Increased IGF2BP1-dependent mRNA stabilization	–	Proliferation, invasion	[126]

^a^ALKBH5 has controversial roles reported in these cancer types

### ALKBH5 as an oncogene

**Fig. 3:**
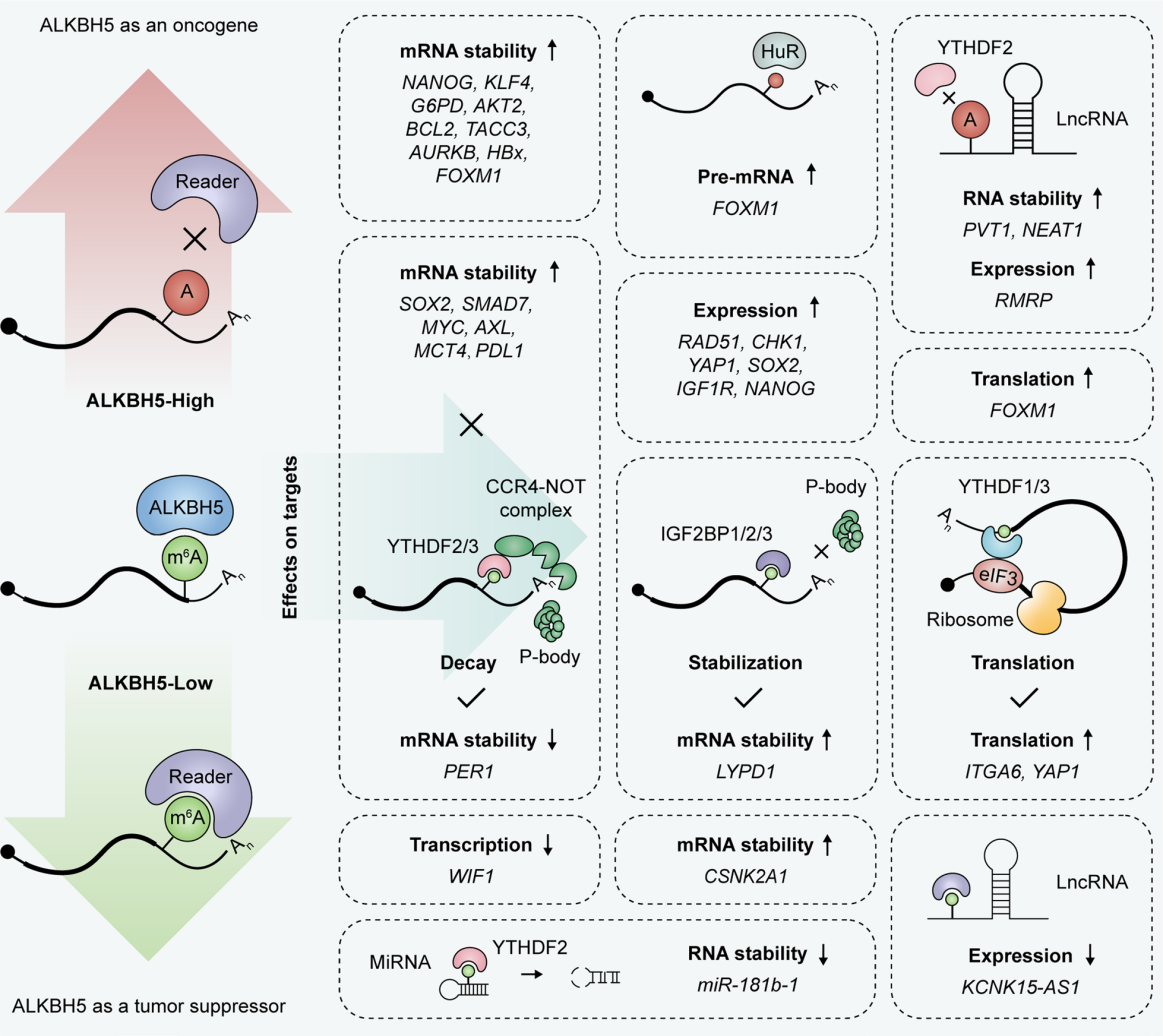
Molecular mechanisms underpinning ALKBH5 regulation on target RNAs in cancers. *HuR* Human antigen R, *YTHDF1/2/3* YTH domain-containing family protein 1/2/3, *IGF2BP1/2/3* Insulin-like growth factor 2 mRNA binding protein 1/2/3

ALKBH5 was shown to induce the formation and development of various malignancies by removing m^6^A modifications on essential RNAs. ALKBH5 overexpression and its contributions to carcinogenesis are presented in Table 2 and Fig. 3, which include the core target RNAs and/or pathways regulated by ALKBH5.

### Breast cancer

Breast cancer represents the major female malignancy, affecting one in 20 women worldwide, with an even higher incidence in high-income countries [70]. ALKBH5 amounts are increased in immortalized and oncogenically-transformed human mammary epithelial cells in comparison with their primary cell precursors [71]. Besides, ALKBH5 is upregulated in breast cancer samples compared to noncancerous tissue [72]. Knockdown of *ALKBH5* inhibits breast cancer cell viability, colony formation and migration, confirming the oncogenic properties of ALKBH5 in this malignancy [72]. In breast cancer, there is a small subset of cells, called breast cancer stem cells (BCSCs), which could both self-renew and differentiate into cancer cells [73]. BCSCs are resistant to chemotherapy and survive the treatment, leading to tumor recurrence and metastasis [73]. Zhang et al. found that NANOG, a core pluripotency factor, is regulated by ALKBH5 in breast cancer cells [74]. Indeed, ALKBH5 overexpression decreases m^6^A methylation of *NANOG* mRNA at 3′ untranslated region (3′UTR), increasing *NANOG* mRNA stability and levels, and induces BCSC enrichment in hypoxic breast cancer cells [74]. Later, these authors found another pluripotency factor, KLF4, is also positively regulated by ALKBH5-mediated m^6^A methylation in breast cancer, contributing to the overall regulation of the BCSC phenotype by ALKBH5 [75].

### Glioma and glioblastoma

Glioma is a central nervous system tumor that seriously endangers the physical and mental health of patients [76]. ALKBH5 is upregulated in glioma and of significance in regulating the metabolism and development of glioma [77]. Specifically, ALKBH5 demethylates the transcript of glucose-6-phosphate dehydrogenase (G6PD), the rate-limiting enzyme of the pentose phosphate pathway (PPP), and enhances its mRNA stability, thus promoting oxidative PPP flux and stimulating the aggravation of glioma [77]. In addition, *AKT2* mRNA was identified as another critical target of ALKBH5 that contributes to the tumor-promoting effects of ALKBH5 in glioma cells [78].

Glioblastoma (GBM) represents the commonest primary brain cancer with high heterogeneity and poor prognosis, making up 54% of all gliomas [79, 80]. GBM stem-cell like cells (GSCs) constitute an important factor in maintaining tumor growth and inducing tumor recurrence [81]. ALKBH5 is overexpressed in GSCs and correlated with reduced patient survival in GBM [82]. It demethylates the nascent transcript of *FOXM1* and promotes the interaction of *FOXM1* pre-mRNA with the nuclear RNA binding protein HuR, resulting in increased FOXM1 expression, which is critical for GSC growth and self-renewal [82]. ALKBH5 also plays an important role in radioresistance and invasiveness of GSCs [83]. ALKBH5 enhances radioresistance by modulating genes involved in homologous recombination (HR) and contributes to the aggressiveness of GBM by upregulating YAP1 expression [83]. Temozolomide, an alkylating agent, is a first-line chemotherapeutic orally administered following surgical excision in GBM [83]. However, temozolomide resistance is frequently found in the middle and late stages of chemotherapy [84]. ALKBH5 could demethylate *SOX2* mRNA, enhancing SOX2 expression and promoting temozolomide resistance in GBM [85].

### Lung cancer

Lung cancer ranks first among cancers in terms of morbidity and mortality globally, with non-small-cell lung cancer (NSCLC) representing the main histological subtype [86]. ALKBH5 undergoes ectopic upregulation in NSCLC, and is tightly associated with reduced patient survival [87]. ALKBH5 promotes proliferation and reduces apoptosis in NSCLC cells by repressing *TIMP3* mRNA stability and translation [87]. *UBE2C*, an oncogene that selectively represses autophagy and promotes cell proliferation in NSCLC, is epitranscriptionally stabilized with reduced m^6^A amounts within its mRNA because of ALKBH5 upregulation in NSCLC [88]. Meanwhile, m^6^A demethylation caused by ALKBH5 stabilizes oncogenic drivers, including SOX2, SMAD7 and MYC, via the m^6^A reader protein YTHDF2 in KRAS mutant NSCLC [89].

In addition to NSCLC, ALKBH5 is elevated in lung adenocarcinoma cells submitted to intermittent hypoxia [90]. ALKBH5 knockout in lung adenocarcinoma cells increases m^6^A abundance in FOXM1 transcript and decreases its translation, leading to reduced cell proliferation and invasion [90]. ALKBH5-dependent m^6^A demethylation of the lncRNA *RMRP* also has an oncogenic effect in lung adenocarcinoma [91]. *RMRP* deficiency in lung adenocarcinoma cell lines suppresses cell proliferation, migration and invasion, and promotes cell apoptosis, mimicking the effects of ALKBH5 inhibition [91].

### Ovarian carcinoma

As a malignant neoplasm affecting women with a high death rate [92], the development ovarian cancer was reported to be tightly associated with m^6^A modification. A study examined the expression profiles of AlkB family members in ovarian serous carcinoma, and revealed that cases highly expressing ALKBH5 have reduced overall survival (OS) and progression-free survival (PFS) [93]. ALKBH5 expression is increased in epithelial ovarian cancer in comparison with noncancerous ovarian tissue [94]. ALKBH5 inhibits autophagy and promotes malignancy in ovarian cancer cells by stabilizing *BCL2* mRNA and promoting BCL2 binding to BECN1 [94]. Besides, ALKBH5-mediated mRNA demethylation increases NANOG expression and enhances aggressiveness of ovarian cancer cells [95].

### Acute myeloid leukemia

Acute myeloid leukemia (AML) represents a fatal hematologic cancer featuring uncontrolled expansion of poorly differentiated myeloid cells, with hematopoietic stem and progenitor cells (HSPCs) retaining their self-renewal ability and impaired myeloid differentiation [96]. Kwok and collaborators demonstrated ALKBH5 is commonly deleted in AML, particularly in individuals with *TP53* mutations, based on the cancer genome atlas (TCGA) data [97], implying ALKBH5’s tumor-suppressive function in AML. However, recent studies showed that ALKBH5 is actually upregulated in human AML, with its elevated amounts associated with reduced patient survival [98, 99]. These studies all demonstrated that ALKBH5 is selectively required for self-renewal of leukemia stem cells (LSCs) but not for normal hematopoiesis [98, 99]. Mechanistically, ALKBH5 enhances malignancy in AML by post-transcriptionally regulating TACC3 and AXL [98, 99].

### Gastric cancer and colon cancer

Gastric cancer (GC) represents a very common malignancy of the digestive tract, ranking fifth and fourth in terms of incidence and mortality among all cancers, respectively [100]. ALKBH5 is overexpressed in GC and downregulates m^6^A levels of the lncRNA *NEAT1*, leading to the upregulation of *NEAT1* expression [101]. Increased *NEAT1* then positively affects the expression of EZH2, a subunit of the polycomb repressive complex, by acting as a scaffold and thus promoting GC invasion and metastasis [101]. Colorectal cancer is the third incident and second deadliest malignancy worldwide, representing about one in 10 cancer cases and deaths [100]. *ALKBH5* knockdown suppresses the proliferative and migratory abilities of colorectal cancer cells partially through ALKBH5-related *NEAT1* downregulation [102], which is consistent with the finding in GC cells [101].

### Other cancers

Besides the above cancers, ALKBH5 is also correlated with poor prognosis and/or plays an oncogenic role in other malignancies, including endometrial cancer [103, 104], renal cell carcinoma [105], hepatitis B virus (HBV) related hepatocellular carcinoma (HCC) [106], osteosarcoma [107], uveal melanoma [108], pituitary adenomas [109], esophageal squamous cell carcinoma [110], cervical cancer [111] and oral squamous cell carcinoma [112]. During the investigation of underlying mechanisms, a set of functional targets of ALKBH5 were identified in these cancers (Table 2; Fig. 3).

### ALKBH5 as a tumor suppressor

While ALKBH5 has overt oncogenic roles in most cancers, it is a tumor suppressor in others (Fig. 2; Table 2). Reduced ALKBH5 amounts has been described in the following malignancies.

### Bladder cancer

Bladder cancer (BC) is among the most prevalent cancers globally, with approximately 573,000 new cases reported in 2020 [100]. Chen et al. demonstrated that ALKBH5 expression in BC shows positive correlations with grade and M1 stage, indicating ALKBH5 might be an oncogene in BC [113]. However, other studies demonstrated that ALKBH5 is actually a tumor suppressor in BC according to cell and animal assays. Yu et al. found that reduced ALKBH5 amounts in BC are associated with decreased patient survival [114]. Functional assays showed ALKBH5 silencing increases the proliferative, migratory and invasive abilities of BC cells and reduces their cisplatin chemosensitivity via a CK2α-related glycolytic pathway [114]. Jin and colleagues examined the role of m^6^A modification in BC, and revealed ALKBH5, but not METTL3, inhibits cell adhesion of BC by repressing ITGA6 expression [115]. Specifically, ALKBH5 decreases m^6^A methylations in the 3′UTR of the *ITGA6* transcript, suppressing ITGA6 protein synthesis mediated by YTHDF1 and YTHDF3 [115].

### Pancreatic cancer

Pancreatic cancer (PC) represents one of the rare cancers whose mortality and incidence rates are almost identical, ranking seventh among deadliest cancers globally [116]. Studies revealed enhanced survival in PC cases highly expressing ALKBH5 [117, 118]. ALKBH5 exerts tumor-suppressive effects via the demethylation of the lncRNA *KCNK15-AS1*, controlling *KCNK15-AS1*-dependent cell migration and invasion in PC [119]. In addition, ALKBH5 post-transcriptionally enhances PER1 expression in an m^6^A-YTHDF2-dependent fashion, activating ATM-CHK2-P53/CDC25C signaling and suppressing PC cell proliferation [120]. Moreover, ALKBH5 acts as a tumor suppressor in pancreatic ductal adenocarcinoma (PDAC) and sensitizes PDAC cells to gemcitabine by reducing *WIF1* mRNA methylation and modulating the Wnt pathway, which leads to the downregulation of C-MYC, Cyclin D1, MMP-2 and MMP-9 [121].

### NSCLC and osteosarcoma

Although most reports showed that ALKBH5 is an oncogene in NSCLC [87–89], as stated above, another work reported its tumor suppressive role in NSCLC. Jin et al. showed ALKBH5 inhibits tumor cell proliferation and metastasis by decreasing YTHDFs-dependent YAP1 expression in NSCLC [122]. Meanwhile, ALKBH5 decreases YAP1 activity by HuR-dependently controlling *miR-107/LATS2* expression [122]. In osteosarcoma, Yuan and co-workers discovered downregulated levels of ALKBH5 in osteosarcoma cell/tissue specimens in comparison with noncancerous osteoblast cell/tissue samples [123], which was contrary to the observation of a previous study [107]. According to Yuan’s study, ALKBH5 overexpression markedly reduces the proliferative, migratory and invasive features of osteosarcoma cells, while triggering apoptosis [123]. Similar to the finding in NSCLC [122], ALKBH5 suppresses osteosarcoma progression via m^6^A-related direct/indirect YAP1 regulation [123]. Specifically, YAP1 expression is directly suppressed through mRNA m^6^A methylation, alongside indirect suppression by ALKBH5-regulated *miR-181b-1* [123].

### Other cancers

ALKBH5 downregulation was demonstrated in clinical colon cancer tissue samples, which was tightly associated with distant metastasis and disease stage [124]. Further functional assays confirmed ALKBH5 overexpression inhibits colon cancer cell invasion in vitro and metastasis in experimental animals [124]. However, the underlying mechanism remains to be elucidated. In addition, ALKBH5 also functions as a tumor suppressor in clear cell renal carcinoma (ccRCC) and HCC. In ccRCC, reduced ALKBH5 gene expression is associated with shorter OS, indicating that ALKBH5 could be used as a prognostic biomarker [125]. In HCC, ALKBH5 suppresses the proliferative and invasive properties of cancer cells by m^6^A-mediated inhibition of LYPD1 [126].

## ALKBH5 and cancer immunity

Recent works have demonstrated that m^6^A modification affects immune response and cancer immunity [127, 128]. As an m^6^A modulator, ALKBH5 have biological significance for both innate and adaptive immune responses. First, ALKBH5 impacts virus infection and antiviral innate immune responses by demethylating the transcripts of genes encoding essential molecules of the innate immune system, including *TRAF3*, *TRAF6*, *MAVSI*, *FNB1* and *OGDH* [58, 59]. Second, ALKBH5-mediated *NR4A1* mRNA demethylation is required for group 3 innate lymphoid cell (ILC3) homeostasis and gut immunity [60]. Third, ALKBH5-dependent upregulation of HMGB1 mediates STING-IRF3 innate immune response in radiation-induced liver diseases [61]. Fourth, ALKBH5 contributes to the regulation of CD4 + T cell homeostasis and function during the induced neuroinflammation by increasing interferon-γ (IFN-γ) and C-X-C motif chemokine ligand 2 (CXCL2) mRNA stability in an m^6^A-dependent manner, leading to enhanced responses of CD4 + T cells and increased recruitment of neutrophils into the central nervous system [62].

Besides, studies have shown that ALKBH5 is involved in the induction of cancer immune evasion. Over the course of cancer development, tumor cells evolve and exhibit multiple mechanisms to inhibit antitumor immune responses and evade tumor immunosurveillance [129]. The expression of inhibitory checkpoint molecules on tumor cells is a major mechanism underlying tumor immune evasion [129]. For instance, programmed cell death 1 ligand 1 (PD-L1) on cancer cells suppress antitumor effector T cells and enables immune evasion through interaction with programmed cell death receptor 1 (PD-1) on T cells [130]. Intrahepatic cholangiocarcinoma (ICC) is a highly aggressive and lethal hepatobiliary malignancy [131]. In ICC, tumor-intrinsic ALKBH5 removes m^6^A modification in *PDL1* mRNA’s 3′UTR and reduces its degradation in a YTHDF2-dependent manner, thus inhibiting cytotoxicity of T cells and mediating immune escape of ICC cells [132].

Apart from tumor-intrinsic ALKBH5–PD-L1 regulating axis, accumulating evidence confirmed the critical role of ALKBH5 in remodeling immune microenvironment in cancer. The immunosuppressive tumor microenvironment (TME) represents another major cause of immune evasion and low immune therapy response in cancer [129]. Regulatory T cells (Tregs) and myeloid-derived suppressor cells (MDSCs) are the dominant subsets of immune cells with immunosuppressive activity in the TME [129]. During Anti–PD-1 treatment for melanoma and colorectal carcinoma, tumor cell expression of ALKBH5 regulates extracellular lactate content and then affects Tregs and MDSCs accumulation in the TME [133]. Specifically, *MCT4*/*SLC16A3*, an ALKBH5 target gene, is responsible for lactate secretion and recruitment of immunosuppressive Tregs and MDSCs [133]. Moreover, tumor-associated macrophages (TAMs), another critical component of the TME, can be recruited and educated by tumor-expressed ALKBH5 under hypoxic conditions [134]. Macrophages are broadly categorized as M1 or M2 types, and TAMs have been shown to exhibit immunosuppressive and tumor-promoting M2-like phenotypes [129, 135]. In glioblastoma multiforme, TAMs are one of the most abundant TME cell types, contributing up to 30% to 50% of brain tumor mass [134, 136]. Through m^6^A demethylation and stabilization of lncRNA *NEAT1*, hypoxia-induced ALKBH5 facilitates paraspeckle assembly and sequesters transcription repressor SFPQ from the *CXCL8*/*IL8* promoter, whose expression and secretion recruit TAMs [134]. In this scenario, tumor-intrinsic ALKBH5 drives TAM infiltration and induces M2 polarization, leading to immunosuppression and tumor progression in glioblastoma multiforme [134].

## Regulation of ALKBH5 expression and function in cancer

**Fig. 4:**
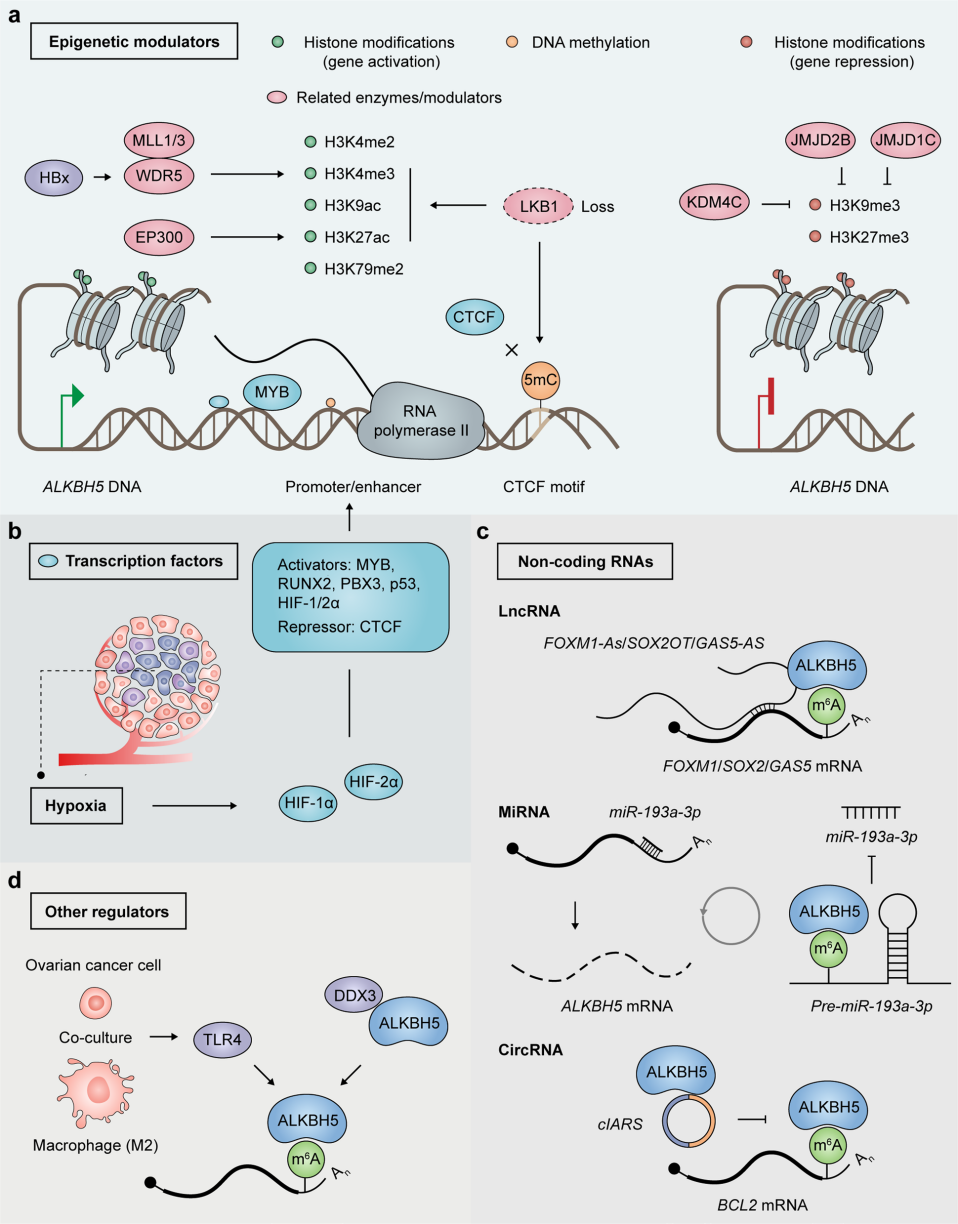
Regulation on the expression and/or function of ALKBH5 in cancers. **a** Epigenetic modulators of ALKBH5. Histone modifications involved in *ALKBH5* transcriptional activation and their modulators (left); LKB1 loss upregulates ALKBH5 by inducing 5mC DNA hypermethylation of the CTCF motif on *ALKBH5*’s promoter, preventing CTCF binding and enhancing active histone modifications (middle); Histone modifications involved in *ALKBH5* transcriptional inactivation and their modulators (right). *HBx* HBV X protein, *MLL* Myeloid/lymphoid or mixed-lineage leukemia protein, *WDR5* WD-40 Repeat Protein 5, *EP300* E1A-associated protein p300, *LKB1* Serine/threonine-protein kinase STK11, *CTCF* CCCTC-binding factor, *KDM4C* Lysine-specific demethylase 4C, *JMJD2B/1C* Jumonji domain-containing protein 2B/1C. **b** Transcription factors of ALKBH5. Transcription activators and repressors bind to promoter or enhancer of *ALKBH5* DNA to control *ALKBH5* transcription; Hypoxia-inducible factors (HIFs) activate *ALKBH5* transcription in response to local hypoxia in cancer. *MYB* Proto-oncogene c-Myb, *RUNX2* Runt-related transcription factor 2, *PBX3* Pre-B-cell leukemia transcription factor 3, *p53* Cellular tumor antigen p53, *HIF-1/2α* Hypoxia-inducible factor 1/2-alpha. **c** Non-coding RNA partners of ALKBH5. Long non-coding RNAs (lncRNAs) function as scaffolds to enhance ALKBH5 binding to their antisense mRNAs; microRNA (miRNA) *miR-193a-3* interacts with 3′UTR of *ALKBH5* mRNA and promotes its degradation, while ALKBH5 demethylates and suppresses *miR-193a-3p* maturation in turn; Circular RNA (circRNA) *cIARS* interacts with ALKBH5 and represses its regulatory effect on *BCL2* mRNA. d Other regulators of ALKBH5. ALKBH5 is positively regulated by TLR4 in ovarian cancer cells after co-culture with M2 macrophages; DDX3 interacts with ALKBH5 and enhances demethylation activity of ALKBH5. *TLR4* Toll-like receptor 4, *DDX3* DEAD box protein 3, X-chromosomal

Increasing research has focused on exploring the mechanisms responsible for the dysregulation of ALKBH5 in cancer. Recent findings suggested that hypoxia, as well as some epigenetic modulators, transcription factors and non-coding RNAs are main contributors to ALKBH5 dysregulation in cancer (Table 2; Fig. 4).

### Hypoxia

Hypoxia, a feature of most tumors, results from fast cell division and abnormal angiogenesis [137]. Hypoxia-inducible factors (HIFs) control transcriptional responses to local hypoxia in cancer and induce tumor progression by affecting cell metabolism, promoting angiogenesis, controlling stem cell state and other cellular processes [137]. HIFs consist of α (HIF-α) and β (HIF-β) subunits and interact with canonical DNA sequences termed hypoxia-response elements (HREs) in the promoters or enhancers of target genes, upregulating 150 genes or more [137]. An early study found that *ALKBH5* is a direct target of HIF-1α, indicating that ALKBH5 may be involved in the regulation of cellular responses to hypoxia [138]. Later, it was demonstrated in human breast cancer cells that ALKBH5 is significantly upregulated under hypoxic conditions, while knockdown of HIF-1α and/or HIF-2α abrogates this effect [74, 75]. Similarly, hypoxia and HIFs elevate ALKBH5 expression in lung adenocarcinoma cells [90], renal cell carcinoma cells [105], endometrial cancer stem cells [104] and pituitary adenoma cells [109], leading to invasive tumor phenotype, enhanced tumor initiation capacity, sustained stem-like state and poor clinical outcomes.

### Epigenetic modulators

Epigenetic modifications, including DNA methylation, histone modifications and nucleosome positioning, affect gene expression with no alteration of the underlying DNA sequence [139]. Dysregulation of these processes is frequently observed in human cancers, some of which are responsible for ALKBH5 dysregulation in cancers.

Histone modifications and associated enzymes are involved in chromatin compaction, nucleosome dynamics and transcription activation or suppression [140]. In leukemia-initiating cells, the promoter regions of ALKBH5 have markedly elevated amounts of active histone markers, including H3K9ac, H3K4me3, H3K4me2 and H3K79me2, and reduced levels of the repressive histone marker H3K27me3 and H3K9me3, leading to a higher degree of chromatin openness [99]. Consistently, a set of H3K4 histone methyltransferases (MLL1 and MLL2) and H3K9 histone demethylases (JMJD2B and JMJD1C) were identified as *ALKBH5* promoter-binding proteins in AML cells [99]. Further investigation confirmed knockdown of *MLL1* and *MLL3*, but not *MLL2* and *MLL4*, markedly reduces ALKBH5 amounts in leukemia cells [99]. Moreover, histone demethylase KDM4C controls ALKBH5 expression in leukemia by reducing H3K9me3 levels and enhancing chromatin accessibility of the *ALKBH5* locus, which promotes the binding of the transcriptional factor MYB and the C-terminal domain (CTD) of the RNA polymerase II to the *ALKBH5* promoter [99]. These results showed ALKBH5 overexpression is controlled by chromatin state in the pathogenesis of AML.

Epigenetic dynamics of ALKBH5 has also been researched in HBV-HCC, uveal melanoma (UM) and NSCLC. The HBV X protein (HBx) was found to induce an elevated degree of epigenetic H3K4me3 modification of chromatin by upregulating WD-40 Repeat Protein 5 (WDR5), contributing to HBV-induced hepatocellular carcinogenesis [141]. It was demonstrated that highly expressed ALKBH5 in HBV-HCC occurs via HBx- and WDR5-dependent H3K4me3 modification of the *ALKBH5* promoter following HBV infection [106]. Elevated ALKBH5 amounts then enhance HBx mRNA expression, forming a positive-feedback loop that contributes to HBV-associated liver carcinogenesis [106]. In UM, EP300-induced H3K27 acetylation activation upregulates ALKBH5 expression [108]. ALKBH5 level is starkly decreased in UM cells after treatment with C646, a histone acetyltransferase suppressor specific to EP300 [108]. Many NSCLC cases contain concurrent *KRAS* mutation, alongside loss of LKB1, which negatively regulates the total 5mC DNA methylation [142–144]. LKB1 deficiency upregulates ALKBH5 by inducing DNA hypermethylation of the transcriptional repressor CTCF binding region on *ALKBH5* gene’s promoter, preventing CTCF binding while enhancing active histone modifications such as H3K4me3, H3K9ac and H3K27ac [89].

### Transcription factors

Transcription factors bind DNA in a sequence-specific manner to control chromatin and transcription, forming a complicated system that impact gene expression [145]. During the investigation of ALKBH5 promoter-binding proteins in AML cells, a set of transcription factors was identified, including RUNX2, MYB and PBX3 [99]. Further investigation confirmed MYB silencing markedly decreased *ALKBH5* mRNA and protein amounts in leukemia cells [99]. The well-known transcription activator P53 is mutated or suppressed in about half of cancers [120]. A TCGA dataset assessment demonstrated elevated and reduced ALKBH5 amounts in the P53 wild-type and mutant groups of PC cells, respectively [120]. P53 interacts with the *ALKBH5* promoter, transcriptionally activating ALKBH5 and indirectly reducing m^6^A amounts in PC cells [120].

### Non-coding RNAs

While ALKBH5 acts as an important factor controlling the expression and functions of a number of ncRNAs, some ncRNAs also influence its expression and function. The crosstalk between ALKBH5 and ncRNAs appears to form a feedback loop with extensive impacts in cancers.

LncRNAs, i.e., noncoding transcripts of more than 200 nucleotides, might play the roles of enhancers, scaffolds or decoys by physically binding other RNAs or proteins [146]. In GBM cells, the lncRNA *FOXM1-AS* (antisense to *FOXM1*) enhances ALKBH5 binding to *FOXM1* nascent mRNA [82]. *FOXM1-AS* suppression mimics ALKBH5 depletion in modulating *FOXM1* methylation and biosynthesis [82]. Similarly, the lncRNA *GAS5-AS* (antisense to *GAS5*) enhances *GAS5* stability by binding to ALKBH5 and controlling m^6^A modifications of *GAS5* in cervical cancer [147]. In GBM cells with temozolomide-resistance, the lncRNA *SOX2OT* functions as a scaffold that recruits ALKBH5, positively regulating its function and expression [85].

MiRNAs are 18–25 nucleotide-long ncRNAs that inhibit gene expression by directly interacting with complementary target mRNAs, which are then degraded or translationally inhibited [148]. *ALKBH5* mRNA was recognized as a *miR-193a-3* target in both glioma and esophageal squamous cell carcinoma (ESCC), with its 3′UTR being a *miR-193a-3p* binding site [78, 149]. *miR-193a-3p* transfection results in substantial ALKBH5 downregulation, while *miR-193a-3p* inhibition upregulates ALKBH5 [78, 149]. Meanwhile, in ESCC, ALKBH5 suppresses the maturation of primary *miR-193a-3p* by decreasing its m^6^A modification in turn, suggesting a feedback regulation between *miR-193a-3p* and ALKBH5 [149].

CircRNAs represent single-stranded, covalently closed RNAs [150]. They interact with and sequester target proteins to adequate subcellular locations, and modulate some protein–protein and protein-RNA interactions [150]. CircRNA *cIARS* (hsa_circ_0008367) controls sorafenib-induced autophagy and ferroptosis in HCC cells through interaction with ALKBH5 [151], which inhibits autophagy by demethylating *BCL2* mRNA and promoting BCL2-BECN1 binding [94]. *cIARS* interacts with ALKBH5 directly and represses its regulatory effect on BCL2-BECN1 autophagy regulatory complex, leading to increased autophagic flux and ferritinophagy [151].

### Other regulators of ALKBH5

DDX3, a DEAD-box RNA helicase, interacts with ALKBH5 in an RNA-independent manner and modulates the demethylation activities of ALKBH5 [152]. The ATP and DSBH domains of DDX3 and ALKBH5, respectively, were required for this interaction [152]. Later, another study confirmed that ALKBH5 is directly regulated by DDX3 in oral squamous cell carcinoma cells [112]. However, the study showed that ATP domain of DDX3 was not essential for DDX3 mediated up-regulation of ALKBH5 [112]. In human ovarian cancer, ALKBH5 and TLR4 amounts are elevated in cancer cells after co-culture with alternatively activated (M2) macrophages [95]. Specifically, TLR4 positively regulates ALKBH5 through NF-κB signaling [95].

## Dual roles of ALKBH5 in cancer

Data from human malignancies, as noted above, suggest a contradictory role for ALKBH5: that of an oncogene in some cancers and that of a tumor suppressor in other cancer types. It should not be a surprising event, considering the fact that ALKBH5 post-transcriptionally regulates a great number of target genes that have distinct functions, either positively or negatively, controlling cell cycle, survival, apoptosis, DNA repair, metabolism, autophagy, and other cellular processes determining the fate of tumor cells. It can be expected that some of those effects might stimulate cell growth, while others might induce negative regulation of cell growth. Thus, the role of ALKBH5 in cancer is mainly dependent on its functional targets in a specific cancer type or cellular context. Theoretically, ALKBH5 may regulate a similar set of target genes in different cells. However, cancer is a complex and heterogeneous disease. The independent studies of different cancer types or studies using different models suggested that the regulatory network of ALKBH5 in cancer varies from case to case. The presence or absence of various protein/RNA partners may account for the variable behavior of ALKBH5 in different cellular contexts.

m^6^A reader proteins are key factors responsible for determining and altering the effect of ALKBH5 on a certain target. For example, YAP1, a transcription co-activator whose activation is important for cancer cell proliferation, survival, migration and invasion [153, 154], is one of the critical targets that mediate ALKBH5’s function in multiple cancers. It was demonstrated that YTHDF1 and YTHDF2 competitively bind m^6^A-modified *YAP1* mRNA to positively and negatively regulate YAP1 expression, respectively [122]. Specifically, YTHDF2 facilitates *YAP1* mRNA degradation through the AGO2 system, whereas YTHDF1 promotes *YAP1* mRNA translation by interacting with eIF3a [122]. In NSCLC, the expression of YTHDF1 is higher in tumor tissues than in normal tissues, while the expression of YTHDF2 is lower in tumor tissues than in normal tissues, indicating that YTHDF1 is more likely to bind to methylated *YAP1* mRNA to promote its translation. As a result, ALKBH5-mediated *YAP1* demethylation negatively regulates YAP1 translation and inhibits the growth of tumor cells in NSCLC [122]. In contrast, ALKBH5 positively regulates *YAP1* mRNA expression in GBM cells, and thus exerts its oncogenic role in GBM [83]. Although the study did not investigate the reader protein of methylated *YAP1* mRNA in GBM, one possibility can be advanced that YTHDF2 is responsible for affecting *YAP1* mRNA stability in GBM cells because numerous studies have shown that YTHDF2 is upregulated in GBM [155–157]. Therefore, potential tissue- or cell-specific differences in m^6^A reader proteins may alter ALKBH5’s regulation of downstream targets and explain the conflicting functions of ALKBH5 in cancers.

The interaction of ALKBH5 with auxiliary ncRNAs is another important aspect of ALKBH5’s regulation on its targets. For instance, lncRNA *FOXM1-AS* functions as a scaffold to increase the ability of ALKBH5 to bind to its antisense mRNA *FOXM1*, providing both tumorigenic and proliferative advantages to cancer cells [82]. In addition, the finding that the interaction of circRNA *cIARS* with ALKBH5 alters the autophagy inhibitory behavior of ALKBH5 may allow us to understand the complexity of this epigenetic regulation [94, 151]. These findings show that cell-specific differences in ncRNAs may alter ALKBH5’s binding affinity for different targets and change the final phenotypic outcome.

Following these possible reasons and examples, it remains difficult to tell clearly what natural cellular contexts allow ALKBH5 to act as an oncogene or tumor-suppressor. However, it is increasingly clear that the effect of ALKBH5 on the cancer cell is determined by the cellular context in which it is present and the functional targets it regulates. Particularly, the presence or absence of other regulatory partners are likely to be critical confounding factors that change the role of ALKBH5 in cancer.

## Therapeutic implications of targeting ALKBH5 in cancer

The findings that ALKBH5 is dysregulated and plays critical roles in multiple cancers support the hypothesis that targeting ALKBH5 can act as an approach to cure different types of cancer. Since ALKBH5 has opposite, context-dependent functions in cancer, approaches to both induce and suppress ALKBH5 could represent viable treatment options for malignancies. For example, ALKBH5 is overexpressed and plays an oncogenic role in many tumors, such as breast cancer [72, 74, 75], Glioma [77, 78, 82] and AML [98, 99]. Thus, pharmacological inhibition of ALKBH5 may exert antitumor effects in these cancer types. In contrast, restoration or augmentation of ALKBH5 expression is a potential therapeutic strategy for the treatment of those cancers in which ALKBH5 plays a tumor-suppressive role, such as bladder cancer [114, 115] and pancreatic cancer [119–121].

Additionally, dysregulation of ALKBH5 was found to mediate drug- or radio-resistance in GBM [85, 158], oral squamous cell carcinoma [112], bladder cancer [114], pancreatic cancer [121] and BRCA-mutated epithelial ovarian cancers [159] (Table 3). Such data suggest that ALKBH5 might serve as a predictive marker for personalized treatment of malignancies, providing insights into overcoming therapeutic resistance in cancer by combining drugs targeting ALKBH5 with chemotherapy, targeted therapy, and/or radiation therapy. Notably, the existence of cancer stem cells with self-renewal ability is a major reason for drug or radio resistance and tumor recurrence [160]. Considering ALKBH5’s functions in cancer stem cell maintenance in breast cancer [74, 75], GBM [82], AML [98, 99] and endometrial cancer [104], ALKBH5 could be a potential target for suppressing cancer stem cells and promoting complete remission in cancer treatment.

**Table 3:** Roles of ALKBH5 in drug- or radio-resistance in human malignancies

Cancer type	ALKBH5 expression	Target RNAs	Target pathways	Therapy	Ref
Glioblastoma	High	*CHK1*, *RAD51*	Homologous recombination (HR) pathway	Radiotherapy	[83]
Glioblastoma	High	*SOX2*	Wnt5a/β-catenin signaling	Temozolomide	[85]
Oral squamous cell carcinoma	High	*FOXM1*, *NANOG*	–	Cisplatin	[112]
Bladder cancer	Low	*CSNK2A1*	Glycolysis pathway	Cisplatin	[114]
Pancreatic cancer	Low	*WIF1*	Wnt signaling pathway	Gemcitabine	[121]
BRCA-mutated epithelial ovarian cancers	Low	*FZD10*	Wnt/β-catenin pathway	PARP inhibitor	[159]
Intrahepatic Cholangiocarcinoma	High	*PDL1*	–	Anti–PD-1 therapy	[132]
Melanoma	High	*MCT4*	–	Anti–PD-1 therapy	[133]

What’s more, immunotherapy, e.g., using antibodies that mediate immune checkpoint blockade, has emerged in recent years as an innovative treatment option for tumors [161]. However, the response rates to immunotherapy for the majority of tumors remain low, due to primary, adaptive, or acquired resistance [161]. ALKBH5 overexpression induces immunosuppressive TME and promotes immune evasion in multiple cancers, including ICC [132], melanoma [133], colorectal carcinoma [133] and glioblastoma multiforme [134], suggesting that ALKBH5 could affect tumor response to immunotherapy. Indeed, ALKBH5 mutation or expression are associated with response to anti–PD-1 immunotherapy in ICC and melanoma (Table 3) [132, 133]. ALKBH5 deletion or inhibition enhances the efficacy of anti–PD-1 treatment in colorectal cancer and melanoma [133], suggesting ALKBH5 could represent a promising therapeutic target to enhance immunotherapy outcome in cancer patients.

## Potential strategies for targeting ALKBH5

**Fig. 5:**
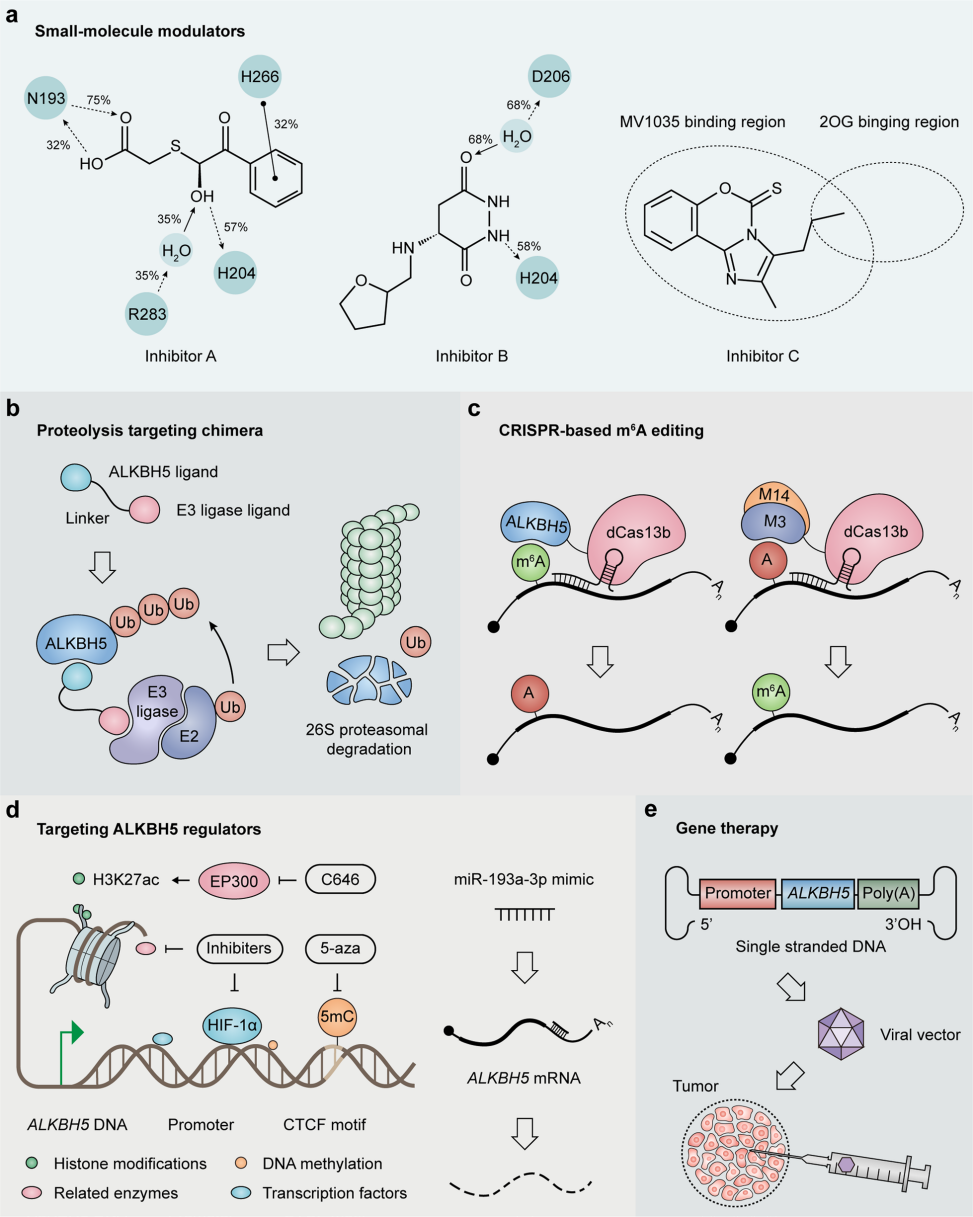
Potential approaches for ALKBH5 targeting in cancers. **a** Small-molecule inhibitors of ALKBH5. **b** Proteolysis targeting chimera (PROTAC) for ALKBH5 protein degradation. **c** CRISPR-based site-specific m^6^A editing systems. *M3* methyltransferase-like protein 3 (METTL3), *M14* methyltransferase-like protein 14 (METTL14). **d** Compounds targeting the upstream regulators of ALKBH5. **e** ALKBH5 gene therapy. *ALKBH5* is delivered within a viral vector followed by direct injection to the tumor

To the best of our knowledge, the reported functions of ALKBH5 in cancer rely on m^6^A demethylation activity in all cases. Potential strategies including small-molecule modulators, proteolysis targeting chimera (PROTAC), programmable m^6^A-editing systems, compounds targeting the regulatory machinery of ALKBH5, as well as gene therapy, could be applied to manipulate ALKBH5-mediated m^6^A demethylation in cancer (Table 4; Fig. 5).

**Table 4:** Strategies for targeting ALKBH5 in cancer

Strategy	Function	Compounds/Methods	Preclinical models	Ref
Small-molecule modulators	Specific inhibitor	2-[(1-hydroxy-2-oxo-2-phenylethyl)sulfanyl]acetic acid	Leukemia and GBM cell lines	[162]
		4-[(furan-2-yl)methyl]amino-1,2-diazinane-3,6-dione	Leukemia and GBM cell lines	[162]
		ALK-04	Murine B16 melanoma model	[133]
	Non-specific inhibitor	MV1035	GBM cell lines	[163]
	Specific activator	Identifying potential compounds by high-throughput screening	–	NR
Proteolysis targeting chimera	ALKBH5 degradation	Linking a ligand of ALKBH5 protein with a ligand of an E3 ubiquitin ligase	–	NR
CRISPR-based site-specific m^6^A editing	m^6^A demethylation	dm^6^ACRISPR	HeLa cells	[171]
	m^6^A methylation	dCas13-M3nls, dCas13-M3M14nes	HEK293T cells	[174]
Targeting ALKBH5 regulators	ALKBH5 suppression	5-azacytidine	Lung cancer cell lines	[89]
		C646	UM cell lines	[108]
		*miR-193a-3p* mimic	Glioma and ESCC cell lines	[78, 149]
	ALKBH5 restoration	Targeting negative regulators of ALKBH5	–	NR
Gene therapy	ALKBH5 restoration	Delivering *ALKBH5* gene by viral vectors	–	NR

*GBM* Glioblastoma, *UM* Uveal melanoma, *ESCC* Esophageal squamous cell carcinoma, *NR* not reported

### Small-molecule modulators

To identify specific ALKBH5 suppressors, a high-throughput in silico screening of 144,000 preselected molecules was performed, with further analysis by the enzyme inhibition assay [162]. The authors ultimately identified two ALKBH5 inhibitors, including 2-[(1-hydroxy-2-oxo-2-phenylethyl)sulfanyl]acetic acid (Fig. 5a, Inhibitor A; IC50 = 0.84 μM) and 4-[(furan-2-yl)methyl]amino-1,2-diazinane-3,6-dione (Fig. 5a, Inhibitor B; IC50 = 1.79 μM), which were tested for activity in leukemia and GBM cell lines [162]. In leukemia HL-60, CCRF-CEM and K562 cells, theses inhibitors blunted cell proliferation in the low micromolar range (IC50 = 1.38–16.5 μM) [162]. However, another leukemia cell line, Jurkat, and glioblastoma A-172 cells had low to no susceptibility, suggesting ALKBH5 suppression by small-molecule inhibitors as a feasible cancer-cell-type-selective anti-proliferative approach [162]. A novel sodium channel blocker, named MV1035 (Fig. 5a, Inhibitor C), potently inhibits ALKBH5 activity by an alternative off-target interaction with ALKBH5, with the binding site of MV1035 in ALKBH5 partially overlapping with that of 2OG [163]. It was demonstrated that the inhibitory effects of MV1035 on glioma cell migration and invasion rely on ALKBH5 inhibition but not sodium channel blocking [163]. Besides, a specific inhibitor of ALKBH5, named ALK-04, could potentiate the efficacy of anti–PD-1 immunotherapy in melanoma treatment, providing a rational for future combination drug therapy [133].

In addition to naturally-derived small-molecule inhibitors of ALKBH5, developing synthetic small-molecule inhibitors represent another approach to inhibit ALKBH5. ALKBH5 requires an interaction with 2OG and ferrous iron to function as an m^6^A demethylase [68]. Therefore, it would be reasonable to design suppressors by taking into consideration the reported binding pocket of this protein. Particularly, a 2OG-tethering approach of concurrently occupying both the 2OG- and substrate-binding sites is of interest. Linking 2OG derivatives with substrate analogs was successfully utilized to develop selective histone demethylase suppressors [164, 165] and AlkB enzyme inhibitors [166], which can be used to design specific ALKBH5 inhibitors in the future.

Given the tumor suppressive role of ALKBH5 in a small number of cancers, activators of ALKBH5 are needed in such cases. However, pharmacologic activators specifically targeting ALKBH5 have not been reported to date, raising the need for developing ALKBH5 activators. The ability to bind metal cofactor (Fe^2+^) and the secondary substrate (2OG) precedes binding of the m^6^A-containing oligonucleotide to ALKBH5 and is crucial for demethylation activity of ALKBH5 [167]. Therefore, the binding sites of Fe^2+^ and 2OG could be used as target areas for identifying potential compounds as activators of ALKBH5 by high-throughput screens. Flavin mononucleotide, a metabolite produced by riboflavin kinase, was found to mediate substantial photochemical demethylation of m^6^A residues of RNAs in live cells, indicating that regulation of m^6^A modification by external small organic molecules is possible [168]. It also provided insights into the development of powerful small molecules as RNA demethylases and specific functional probes targeting m^6^A modification for biomolecular and treatment purposes [168].

### Proteolysis targeting chimera

Besides small molecule-based occupancy-driven pharmacology as an approach to inhibit ALKBH5 functions, PROTAC represents a new technological method for degrading proteins of interest (Fig. 4b). A PROTAC is a heterobifunctional molecule that consists of a protein comprising a ligand for interacting with a target protein and another interacting with an E3 ubiquitin ligase, with a linker utilized for connection (Fig. 5b, right panel) [169, 170]. Indeed, a PROTAC recruits the E3 ligase to the protein of interest and induces its ubiquitination and degradation [169, 170]. The main advantages of PROTACs include their high selectivity, the potential to target non-druggable proteins, and the capacity of overcoming resistance to small-molecule suppressors via targeting of mutant proteins [170]. Using PROTACs for degrading proteins critical for tumorigenesis represents a potential therapeutic approach in cancer. PROTACs have be successfully used in solid (targeting AR, ER, FAK and P38) and hematological (targeting BRD4, BTK, BCR-ABL and CDK-6) tumors [170]. Importantly, the first PROTACs against AR and ER have been applied clinically [170]. Whether such approach would degrade ALKBH5 in cancer deserves further attention.

### CRISPR-based site-specific m^6^A editing

A potential concern is that targeting ALKBH5 utilizing the abovementioned techniques or reagents would affect the global demethylation of mRNAs, including both oncogenes and tumor suppressor genes, or genes with other functions, which may lead to potential deleterious effects [171]. According to previous studies (Table 2; Fig. 3), the tumor-promoting or suppressing role of ALKBH5 is largely mediated by a few critical targets, suggesting that modulating the m^6^A on specific RNAs could alleviate tumor progression with reduced side effects. Therefore, developing a site-specific m^6^A modulation system may provide a more potent tool for correcting dysregulated m^6^A modifications in cancers.

The development of Cas13, a novel RNA-targeting CRISPR-associated nuclease, enables the targeting of dynamics of endogenous RNAs [172, 173]. Li et al. have recently developed a CRISPR–Cas13b-based tool, named dm^6^ACRISPR, to specifically demethylate target mRNAs [171]. The dm^6^ACRISPR fusion protein was constructed by linking catalytically inactive Cas13 enzyme (dCas13b) to ALKBH5 (Fig. 5c, left panel) [171]. dm^6^ACRISPR effectively demethylated target transcripts without significant off-target effects, and was successfully applied to target oncogenic mRNAs, such as *EGFR* and *MYC* mRNAs, to decrease HeLa cell proliferation [171]. In addition, another team designed programmable m^6^A modification utilizing the CRISPR-based METTL3 methyltransferase domain and METTL3:METTL14 methyltransferase complex, named dCas13-M3nls and dCas13-M3M14nes respectively, which can install m^6^A on targeted mRNAs (Fig. 5c, right panel) [174]. Together, these technologies could work together to add or delete functional essential m^6^A sites on specific RNAs. Considering that CRISPR-based gene therapy has achieved great success, with many ongoing phase I/II clinical studies [175], CRISPR-based m^6^A targeting may provide a powerful strategy for m^6^A-based precision medicine.

### Targeting ALKBH5 regulators

The finding of upstream regulators of ALKBH5 in cancers (Table 2; Fig. 4) provides an alternative approach to inhibit ALKBH5 by targeting its regulatory machinery. These ALKBH5 regulators, including multiple epigenetic modulators, transcription factors and non-coding RNAs, are also dysregulated in specific cancers and exert similar effects on cellular phenotypes as ALKBH5. Inhibitors of these regulators, like DNA methylation inhibitor 5-azacytidine (5-aza) [89] and histone acetyltransferase EP300 inhibitor C646 [108], as well as miRNA *miR-193a-3p* mimic [78, 149] are able to indirectly or directly suppress ALKBH5 expression in cancer cells (Fig. 5d). Numerous epigenetic, HIF-targeted and miRNA-directed therapies have been examined in clinical trials [176–178], showing potential of targeting these kinds of molecules for cancer treatment, which might exert anti-tumor effects partly through altering ALKBH5-mediated m^6^A demethylation.

In the setting that ALKBH5 functions as a tumor suppressor, inhibiters targeting negative regulators of ALKBH5 demonstrate great pharmacological potential as cancer remedy. This approach has proven to be beneficial in the case of activating tumor suppressor p53 for cancer treatment. Several compounds have been developed to specifically target MDM2, a negative regulator of p53, leading to p53 stabilization and activation [179]. For ALKBH5, epigenetic modulators, e.g., protein arginine methyltransferase PRMT7, that function to repress *ALKBH5* transcription have been investigated with mechanistic insights gained. PRMT7 binds to the BRG1-related hSWI/SNF chromatin remodeling complex and carries out the methylation of histones H2A Arg-3 (H2AR3) and H4 Arg-3 (H4R3), leading to negative regulation of ALKBH5 expression [180]. Thus, inhibitors targeting PRMT7 may effectively reverse the inhibition of ALKBH5 transcription. In addition, SUMO E2 conjugation enzyme UBC9 and E3 ligase PIAS4 mediates ALKBH5 SUMOylation at lysine residues K86 and K321, which markedly inhibits demethylase activity of ALKBH5 by blocking its binding to m^6^A RNA species [57], indicating the design of small-molecule inhibitors targeting the modulators of ALKBH5 post-translational modifications might be another intriguing strategy to reactivate ALKBH5. Further understanding of regulatory mechanisms on the expression, activation and stabilization of ALKBH5 may provide more ideal targets to up-regulate its level and enhance its tumor-suppressive activities in some cancers.

### Gene therapy

Gene therapy is the treatment of a genetic disease by repairing or reconstructing defective genetic material [181]. For cancers caused by a deficiency in ALKBH5, such as bladder cancer [114, 115] and pancreatic cancer [119–121], the most direct way to restore the expression and function of ALKBH5 is to introduce wild-type *ALKBH5* into cancer cells. This can be achieved by in vivo delivery of ALKBH5 gene into the target tumor cells by vectors based on retroviruses, adenoviruses or adeno-associated viruses (AAVs) [181]. Due to their gene delivery efficacy, lack of pathogenicity, and strong safety profile, AAV vectors may be an excellent platform for delivering therapeutic genes [182]. Recombinant AAV vectors are generated by replacing the endogenous rep and cap genes with an expression cassette consisting of a promoter driving a transgene of interest and a poly(A) tail (Fig. 5e) [183]. The viral vectors can be administered intratumorally and are able to infect cancer cells to achieve ectopic expression of the therapeutic transgene [183]. AAV vector-mediated gene delivery was recently approved for the treatment of inherited blindness and spinal muscular atrophy [183, 184]. Additionally, AAV vectors have been increasingly employed in a variety of preclinical tumor models to deliver a wide array of therapeutic genes and tumor suppressor genes, such as *TP53* [182]. The application of gene therapy to restore ALKBH5 expression in cancers as well as its clinical efficacy and biosafety await further validation.

## Conclusion and perspectives

As one of the m^6^A regulators, ALKBH5 has a critical function in keeping the balance between methylation and demethylation of RNAs. It is well established that ALKBH5-mediated post-transcriptional regulation of gene expression is crucial for both normal physiological and pathophysiological events. Studies that implicate ALKBH5 in the pathogeneses of multiple malignancies have emerged at a rapid pace in recent years, providing novel insights into ALKBH5’s functions. ALKBH5 could modulate the epitranscriptome in cancer, causing alterations in cell proliferation, survival, invasion and metastasis, drug sensitivity, cancer stem cell state and cancer immunity. However, there remain some issues to be addressed. First, it is noticeable that in some cancers, including NSCLC [87–89, 122], colon cancer [101, 102, 124], renal cell carcinoma [105, 125] and osteosarcoma [107, 122, 123], ALKBH5 plays oncogenic and/or tumor-suppressive roles, which might result from tumor heterogeneity and/or various models. Thus, a comprehensive investigation is required for exploring the exact context in which ALKBH5 promotes or inhibits carcinogenesis, especially for those cancers in which the role of ALKBH5 is highly contentious. Second, it is reasonable that m^6^A methyltransferases and demethylases exhibit opposite effects in a given pathology. However, exceptions have been reported. For instance, both m^6^A writers and erasers, including METTL3, METTL14, FTO and ALKBH5, are aberrantly upregulated, with major oncogenic functions in AML [98, 99, 185–187]. How enzymes with opposite functions play similar roles in a given cancer deserves further investigation. Third, recent studies showed that many m^6^A regulators, such as METTL3 [35, 36, 188] and VIRMA [189, 190], function in cancers independently of catalytic activity. However, m^6^A-independent functions of ALKBH5 and their potential roles in cancer remain unknown, representing another research direction for future study.

Given the functional importance of ALKBH5 in multiple malignancies, targeting dysregulated ALKBH5 is an attractive approach for treating cancer. ALKBH5 has been shown to exhibit strict substrate specificity by only demethylating m^6^A on ssRNAs [21], and its modulation on m^6^A modification is reversible, making it an ideal therapeutic target. To date, studies have identified a few inhibitors of ALKBH5. However, issues with potency, selectivity and cytotoxicity need to be addressed. In addition, CRISPR-based m^6^A targeting tools have been developed to achieve site-specific m^6^A modulation of target oncogenes or tumor suppressors. Besides, PROTACs, gene therapy, as well as inhibitors against regulators of ALKBH5 could also be considered as efficacious therapeutic candidates. Remarkably, several companies have successfully developed small-molecule suppressors of METTL3 and FTO, with high efficacy and selectivity [191–195], and some of them have yielded encouraging preliminary findings in preclinical investigations. Due to the recent finding of ALKBH5 as an m^6^A eraser and its roles in cancer, targeting of ALKBH5 for clinical application is much less examined than METTL3 and FTO, and is still in its infancy. The discovered differences in 2OG binding pockets between ALKBH5 and FTO could help develop selective compounds targeting these two RNA demethylase. Indeed, crystallographic and biochemical analyses applying multiple 2OG analogs demonstrated ALKBH5’s active site cavity is starkly smaller compared with FTO’s, with ALKBH5 preferentially binding small-molecule inhibitors [67, 69]. Collectively, continued efforts are required for designing and optimizing approaches for ALKBH5 targeting in cancer therapy.

In conclusion, we summarized the recently described critical functions and therapeutic potential of ALKBH5 in cancer. The therapeutic targeting of ALKBH5 is just in its early stage. With increased knowledge of ALKBH5 structure, mechanisms behind ALKBH5-mediated carcinogenesis and drug response, alongside comprehensive preclinical analysis, ALKBH5-targeting therapeutics may be applied clinically in the future.

## Data Availability

Not applicable.

## Abbreviations

m^6^A: N^6^-Methyladenosine
NGS: Next-generation sequencing
rRNA: Ribosomal RNA
miRNA: MicroRNA
lncRNA: Long non-coding RNAs
circRNA: Circular RNA
snRNA: Small nuclear RNA
MTC: Methyltransferase complex
FTO: Fat mass and obesity-associated protein
ALKBH5: Alpha-ketoglutarate-dependent dioxygenase alkB homolog 5
ncRNA: Non-coding RNA
2OG: 2-Oxoglutarate
DSBH: Double-stranded β-helix
m^6^Am: N^6^,2-O-dimethyladenosine
m^1^A: N^1^-methyladenosine
m^3^T: 3-Methylthymine
ssDNA: Single-stranded DNAs
m^3^U: 3-Methyluracil
ssRNA: Single-stranded RNA
NRL: Nucleotide recognition lid
BCSC: Breast cancer stem cell
3′UTR: 3′ Untranslated region
G6PD: Glucose-6-phosphate dehydrogenase
PPP: Pentose phosphate pathway
GBM: Glioblastoma
GSC: Glioblastoma stem-cell like cell
HR: Homologous recombination
NSCLC: Non-small cell lung cancer
OS: Overall survival
PFS: Progression-free survival
AML: Acute myeloid leukemia
HSPC: Hematopoietic stem and progenitor cell
TCGA: The cancer genome atlas
LSC: Leukemia stem cells
GC: Gastric cancer
HBV: Hepatitis B virus
HCC: Hepatocellular carcinoma
BC: Bladder cancer
PC: Pancreatic cancer
PDAC: Pancreatic ductal adenocarcinoma
ccRCC: Clear cell renal carcinoma
ILC3: Group 3 innate lymphoid cell
IFN-γ: Interferon-γ
CXCL2: C-X-C motif chemokine ligand 2
PD-L1: Programmed cell death 1 ligand 1
PD-1: Programmed cell death receptor 1
ICC: Intrahepatic cholangiocarcinoma
TME: Tumor microenvironment
Treg: Regulatory T cell
MDSC: Myeloid-derived suppressor cell
TAM: Tumor-associated macrophage
HIF: Hypoxia-inducible factor
HRE: Hypoxia-response element
H3K9ac: Acetylation of lysine 9 on histone 3
H3K4me3: Tri-methylation of lysine 4 on histone H3
H3K4me2: Dimethylation of lysine 4 on histone H3
H3K79me2: Dimethylation of lysine 79 on histone H3
H3K27me3: Tri-methylation of lysine 27 on histone H3
H3K9me3: Tri-methylation of lysine 9 on histone H3
CTD: C-terminal domain
UM: Uveal melanoma
HBx: HBV X protein
WDR5: WD-40 Repeat Protein 5
H3K27ac: Acetylation of lysine 27 on histone 3
ESCC: Esophageal squamous cell carcinoma
PROTAC: Proteolysis targeting chimera
5-aza: 5-Azacytidine
H2AR3: Histone H2A Arg-3
H4R3: Histone H4 Arg-3
AAV: Adeno-associated virus
